# Empirical Insights into Eye-Tracking for Design Evaluation: Applications in Visual Communication and New Media Design

**DOI:** 10.3390/bs14121231

**Published:** 2024-12-21

**Authors:** Ruirui Guo, Nayeon Kim, Jisun Lee

**Affiliations:** 1 Department of Communication Design, Changzhou University, Changzhou 213159, China; grr8201@cczu.edu.cn; 2 Department of Spatial Design and Consumer Studies, The Catholic University of Korea, Bucheon 14662, Republic of Korea; 3 School of Planning, Design and Construction, Michigan State University, East Lansing, MI 48824, USA; leejisu4@msu.edu

**Keywords:** eye-tracking technology, design evaluation, visual attention, consumer behavior, visual communication, new media design

## Abstract

(1) Background: As digital technology continues to reshape visual landscapes, understanding how design elements influence customer experience has become essential. Eye-tracking technology offers a powerful, quantitative approach to assessing visibility, aesthetics, and design components, providing unique insights into visual engagement. (2) Methods: This paper presents a systematic review of eye-tracking methodologies applied in design research. Thirty studies were selected for analysis from recognized academic databases using the Preferred Reporting Items for Systematic Reviews and Meta-Analyses (PRISMA) method. Employing the Population, Intervention, Comparison, and Outcomes (PICO) framework, this review focused on experimental studies in visual communication and new media design that utilized visual symbols for communication and leveraged new media technologies. (3) Results: The findings corroborated that eye-tracking technology offers in-depth insights into gaze patterns, visual perception, and attention, which can inform design strategies. This review shows that assessing visual designs based on eye-tracking data can enhance consumer-centered interfaces, better align with user preferences, and foster more engaged behaviors in both digital and physical environments. (4) Conclusions: This review deepens our understanding of the cognitive and emotional processes underlying visual engagement. It also suggests new avenues for integrating diverse eye-tracking metrics into design evaluation, offering practical applications for improving design strategies and advancing the field of design research.

## 1. Introduction

The utilization of eye-tracking technology in design evaluation research has garnered significant attention due to its capability to capture visual responses and attentional processes. In design studies, capturing visual attention is crucial for understanding consumer behavior and subsequently optimizing consumer interactions and design effects [1]. As customer experience plays a significant role in shaping consumer behavior [2,3], good or bad consumer experience has a direct effect on product usage and sales [4]. Moreover, consumer behavior shapes customer preferences, perceptions, and decision-making processes [5].

Eye-tracking technology is an innovative, non-invasive cognitive neuroscience technology that can capture and record the process of consumers’ eye movements when browsing product interfaces to form objective data [6]. It has become an effective method for analyzing consumer information processing and revealing consumer behavior. 

Cognitive psychology research has found that visual attention can reflect the cognitive processing in the brain [7]. The cognitive processing theory explains that individuals’ cognitive processing affects their response to information [8]. Consumers who devote more visual attention to a product have more positive product preferences that affect their consumption decisions [9]. Thus, as technology advances, it is important to quantitatively evaluate the influence of design elements on customer experience.

While traditional design evaluation methods such as surveys and self-reports are subject to inherent limitations in capturing the unconscious aspects of consumer behavior, eye-tracking technology offers an objective and non-intrusive approach to investigating customer attention and visual perception [4]. By precisely tracking eye movements, fixations, and gaze patterns, eye-tracking allows researchers to gain profound insights into the cognitive processes involved in design evaluation [10]. Eye movements provide evidence of engagement in cognitive processes [11]. Specifically, increased fixation duration has been associated with increased cognitive effort, and a longer gaze indicates interest or confusion [12].

Despite the growing interest in eye-tracking technology, there are still significant gaps in research reviews related to design, particularly systematic reviews in the fields of visual communication design, new media design, and recent innovations, such as AI-driven eye-tracking or real-time design adjustments. Furthermore, most eye-tracking experiments related to design research focus heavily on gaze and fixation metrics, often overlooking other key eye-tracking measures, such as saccades, pupil dilation, and blinks, which could offer deeper insights into emotional responses and cognitive processing. A saccade is defined as the rapid, involuntary movement of the eyes when shifting gaze from one point to another. It can reveal visual search strategies and attention allocation [13]. Pupil dilation is the dilation of the pupil, which can reflect psychological excitement [14]. Blinks refer to the rapid closing and opening of the eyes. By recording the frequency and duration of blinking, researchers can infer the attention state and fatigue level of a subject [15]. 

Furthermore, the broader application of eye tracking across different design domains remains underexplored. The eye movement and pupillary response in relation to emotional or cognitive processing provide valuable information for one’s higher cognitive function and state of affect [16] and provide a more objective understanding of cognitive feedback for design practice [12]. In the realm of design practice, the underlying interests of consumers should be understood, and a positive relationship between the product and the consumer should be maintained to help companies make informed decisions before finalizing the design. 

The application of eye-tracking technology in design research makes it possible for researchers to uncover the cognitive mechanisms that influence consumers’ perception and evaluation of design, which also enables the identification of visual elements that capture attention and elicit emotional responses through precise tracking of consumers’ eye movements, fixations, and gaze patterns [14]. However, comprehensive reviews that consolidate eye-tracking applications in design are lacking, particularly in areas like visual communication and new media design, creating a need for a systematic overview of current research.

Eye-tracking technology significantly enhances the applicability in design, providing researchers with the means to analyze consumers’ visual exploration of both physical and digital displays. By tracking eye movements, designers can evaluate the visibility and effectiveness of display elements, optimize visual merchandising strategies, and direct consumers’ attention toward essential product information. A previous eye-tracking study by Chiu et al. (2023) confirmed that reusable packaging and monotone logos attract consumers’ attention more quickly than original packaging [17]. 

Despite advancements in design technology, comprehensive reviews that bridge the gap between traditional design evaluations and modern tools like eye tracking, particularly in areas like visual communication and new media, are lacking. This systematic review addresses this gap by providing a consolidated view of eye tracking’s application in these design domains, identifying trends, and suggesting future research avenues. Specifically, this study investigated the application of eye-tracking technology in design research to gain insights into the interaction between consumer behaviors and design elements. 

The focus was on the potential benefits of integrating eye-tracking data into visual communication design, including designs that use visual symbols for communication and new media designs that rely on emerging media technologies. The primary research scopes were to (i) analyze research papers using the Population, Intervention, Comparison, and Outcomes (PICO) framework commonly employed in evidence-based practice; (ii) review the various types of eye-tracking technology utilized in design research; (iii) explore how design evaluation is carried out using eye-tracking technology; and (iv) discuss the limitations and considerations of using eye-tracking technology in design research, as well as potential future directions for integrating this technology with other interdisciplinary approaches.

Ultimately, this review explores the potential applications of eye-tracking technology in design evaluation, addresses the methodological challenges associated with its use in design research, and outlines future research directions to advance the field. By identifying gaps in the literature and promoting interdisciplinary and integrative approaches, this review aims to consolidate existing knowledge while enhancing both the theoretical understanding and practical applications of eye tracking across diverse design disciplines.

## 2. Eye-Tracking Technology

The inception of eye-tracking technology dates back to the early 20th century, with the first devices consisting of specialized contact lenses affixed to a pointer [18]. Early research into eye-tracking technology, which sought to understand the most basic assumptions about how the brain and visual system work together, was often solely academic, too complex, and expensive to apply commercially [4]. In the 1940s, a system for tracking eye movements with film recordings was developed with the goal of systematically studying user interface interaction to improve system design [19]. In the 1960s and 1970s, video-based eye trackers appeared—a change that ushered in a new era of eye tracking and facilitated the development of other aspects of eye-tracking technology [4]. Since the 1970s, eye-tracking technology has been employed in diverse domains, including medicine, education, and marketing [18,20]. By the end of the 1990s, breakthroughs in the design of hardware and software for eye tracking broadened its applicability beyond academia, enabling its use in various commercial customer experience laboratories [4].

Improvements in eye-tracking technology have made eye-tracking more affordable and user-friendly for both participants and researchers. Video-based eye trackers can determine the direction of gaze with a high degree of accuracy by measuring the position of the corneal reflection of infrared light relative to the pupil. These can be found in both table and head-mounted configurations and allow for eye tracking in real-time, enabling a much wider range of experimentation than was previously possible [21]. As a result, the use of eye tracking in research across a wide range of disciplines has exploded [21].

In design research, three primary categories of eye-tracking systems exist: screen-based, wearable, and VR-integrated systems. Screen-based eye trackers come in three types—desktop, stationary, and remote. While the basic concepts of each type are the same, the processes and interpretations involved can be varied and complex. Screen-based eye-tracking devices, which are more commonly used for non-invasive eye tracking, must be calibrated before each use and mounted on a computer or screen. Stationary eye trackers are ideal for use in the laboratory [21]. While evaluating the design, the subject must be seated in front of a screen-mounted device that can record observations of any screen stimulus or offline stimuli. Some trackers require a chin rest to stabilize the head, as the chin rest can improve the accuracy of the measurement [21].

Next, a wearable eye tracker is mounted on the lightweight eyewear, which allows the evaluator to move freely while continuously monitoring their eye movements and capturing their visual experience. Unlike screen-based eye-tracking devices, wearable eye trackers do not require head positioning or pre-calibration. These devices use wireless connections to transfer data to a computer or cloud storage. Eye-tracking glasses, much like traditional eyeglasses, are suitable for use in dynamic environmental settings such as driving studies and visual perception investigations in indoor/outdoor environments.

Finally, the convergence of eye-tracking technology and recent research has facilitated the integration of eye tracking with virtual reality (VR) [22], making it possible to detect and analyze the user’s eye movements in a VR environment. VR eye tracking has several benefits. It allows customers to experience immersive sensations in highly controlled environments, provides more in-depth information about the assessor’s behavior, and helps define the area observed by the assessor more easily. With eye-tracking technology, designers can monitor the user’s focus in a VR environment in real-time, thereby optimizing the spatial design layout and material selection. This technology can help designers quickly identify and solve potential problems, avoiding unnecessary waste and delays during actual construction. Eye-tracking technology can also simulate the effects of different materials and present them to designers in real-time. Through virtual reality environments, designers can intuitively perceive the texture, color, and gloss of different materials to better select and match materials and enhance the beauty and quality of the building. Therefore, the use of VR eye-tracking technology offers considerable potential for conducting effective architectural and spatial design assessments and establishing a platform to study various environments beyond a single geographic location [23].

## 3. Methods

### 3.1. Paper Selection Criteria

A literature review, coupled with the Preferred Reporting Items for Systematic Reviews and Meta-Analyses (PRISMA) framework, served as the foundation for identifying pertinent studies in this research, with the goal of assessing the potential of employing eye-tracking techniques in design evaluation. 

The search strings employed were limited to papers presented in written English and published between 2010 and 2023 in international journals. This timeframe aligns with the contemporary classification of design domains and recent advancements in eye-tracking technology. We included only studies from articles in peer-reviewed journals and excluded conference proceedings and dissertations, as the latter typically represent initial experiments and have smaller sample sizes, leading to a potentially weaker data analysis. The inclusion criteria were limited to experimental studies involving eye tracking in the context of design. The selected papers were deliberately employed to guarantee the incorporation of eye-tracking technology within the framework of experimental design studies and rigorous data analysis. 

We excluded review papers and case studies related to eye-tracking technology. The keywords utilized for this purpose were eye tracking, design evaluation, visual communication design, new media design, customer experience, and visual attention (Table 1). Search queries were conducted across reputable databases, including Web of Science, Scopus, and Science Direct, using the terms “eye tracking” AND “design evaluation” AND “visual communication design” AND “new media design” AND “customer experience” AND “consumer behavior” AND “visual attention.” The Boolean operator “AND” was utilized to explore broader categories, while “OR” was employed for the investigation of subcategories. We excluded non-experimental papers that were not related to design and eye tracking, had no comparators, and did not respond to the PICO criteria. Additional papers were identified through subjective inspection of the reference lists of relevant publications. 

These criteria ensured the reliability and relevance of the summary results, focusing on the evaluation and application of eye-tracking technology in visual communication design and new media design, and providing a valuable reference for future design research. According to Yi (2021), visual communication design utilizes visual symbols to convey information, encompassing typography, logo, illustration, advertising, packaging, display, and film and television design. New media design, on the other hand, refers to interactive media characterized by two-way communication, relying on computer technology to enhance interactivity and performance [24].

A total of 432 studies were identified through these database searches, of which 96 were initially excluded because they did not meet our paper selection criteria. Of the remaining articles, 118 duplicates were excluded. In the next phase, the inclusion and exclusion criteria were applied to the titles and abstracts (Table 2).

**Figure 1:**
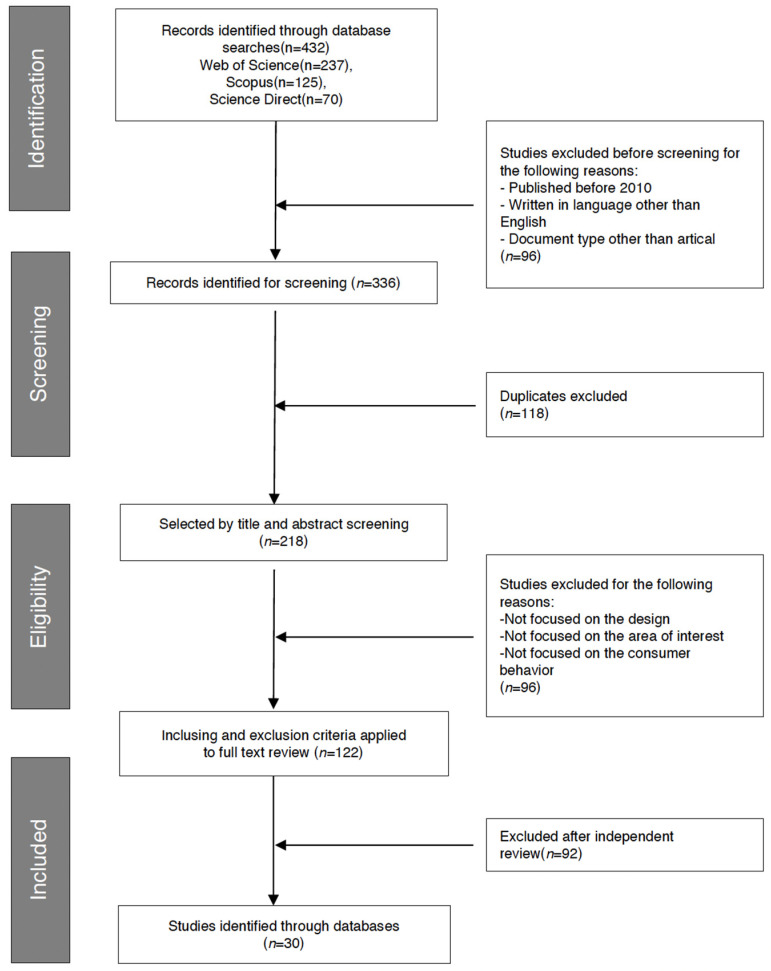
PRISMA Flow Diagram.

A detailed full-text review of 122 articles was then conducted to confirm the eligibility criteria. Ultimately, 30 eligible studies were included in the final analysis (Figure 1).

### 3.2. Analysis Methods

This review utilized the analysis methods suggested by the Cochrane Handbook for Systematic Reviews of Interventions and psychometric tools for assessing communication styles (CSI-B/I) [25]. Papers were categorized and analyzed according to the PICO framework of Higgins et al. (2019) [26]. The PICO criteria in the selected papers were reviewed. Population (P) refers to participant characteristics such as sample size, age, gender, and condition; Intervention (I) refers to tested variables; Comparison (C) denotes conditions of comparison within each group of independent variables; and Outcome (O) refers to the results of consumer psychological measurement data, such as consumer behavior, psychological perception, and attention, obtained using eye-tracking technology. The 30 studies selected for this study are shown in Table 3. 

## 4. Results

### 4.1. Results of Analysis Using PICO Framework

#### 4.1.1. Population

The participant sample sizes in the analyzed studies ranged from a minimum of 20 to a maximum of 255 individuals, as shown in Table 4. The subjects involved in the studies were aged between 18 and 65 years. Regarding gender, five studies did not specify the gender of the participants and were thus excluded from this analysis. Among those included, 37.8% of participants were identified as male, while 62.2% were identified as female.

A sizable proportion of evaluators involved in the 30 studies were college students and faculty members, indicating that they had relatively high levels of education. All participants were normal-sighted. Some evaluators were local town residents and design students. Local urban residents have a deeper understanding of the local culture and environment, which enables them to provide feedback that is more relevant to the research objectives. Design students usually have a certain theoretical knowledge of aesthetics and design, which enables them to provide more professional and in-depth feedback. Other participants were recruited based on specific skills, such as those with experience in marketing activities, MBA programs, or extensive work experience.

#### 4.1.2. Intervention and Comparators

The selected studies employed a range of interventions in visual communication and new media design, focusing specifically on visual stimuli and web-based interfaces. Interventions in visual communication design include elements such as product packaging, advertisement type, and layout configuration. For example, some studies have examined variations in product packaging by comparing label illustration styles, packaging materials, and layout features, such as the arrangement of logos and product images. These interventions aimed to uncover how specific visual features attract and sustain consumer attention. Comparators typically involve alternative designs highlighting key variations (e.g., additive-free vs. standard yogurt types and gender-specific vs. neutral advertisements), enabling a clearer assessment of consumer preferences and attention patterns relative to each design variation.

In new media design, interventions are centered on the interface layout, navigation, and page complexity. Studies have frequently assessed consumer reactions to different web page layouts (e.g., list vs. matrix display modes, simple vs. complex designs) and specific interface features, such as primary navigation novelty, to evaluate their effects on user engagement and cognitive load. Comparators in these cases involved alternative configurations that allowed for the direct observation of differences in user attention and engagement, particularly when navigating complex multimedia environments or interactive platforms. Stimuli for these comparisons typically included screenshots or interactive interfaces closely resembling real-world web layouts, providing ecological validity to the findings and supporting practical applications in digital marketing and user interface design. Table 5 summarizes the interventions and comparators used in the selected studies.

#### 4.1.3. Outcome

Table 6 illustrates the tools and measurement metrics used in the eye-tracking experiments. SMI and Tobii are the most widely used eye-tracking instruments for measuring the number of gazes and gaze duration. Because it is generally considered that gaze is a good indicator of attention, gaze and gaze duration are the basic units of visual information processing [56]. Gaze duration reflects the length of time spent processing information, which is important for understanding the information processing process. More advanced assessments of parameters, such as pupil shape, corneal boundaries, and pupil dilation, were also included in this analysis. 

Understanding consumer behavior through visual attention has been an important focus of studies. In Ballco’s research [27], different types of yogurt were compared using eye-tracking technology metrics, such as gaze duration and gaze count. The study found that the likelihood that a product will be purchased increases with greater consumers’ visual attention to a product. Based on these data, the study further revealed a positive relationship between visual attention and consumer choice behavior. Boscolo [30] further explored the influence of gender on differences in visual attention and attitudes toward advertising. By setting the average total duration of gaze as an indicator, the study compared the responses of different genders to visual elements. The results showed that the production of gender-specific or gender-neutral advertising can affect the audience’s visual attention and emotional response, which can subsequently affect products and brands. Modi [1] compared and analyzed web design, search options, and page layout by fixing eye-tracking metrics, counts, and first focus times. The results show that the interface layout of e-commerce portals has a significant effect on customer decision-making behavior during online shopping. Sophie Lacoste-Badie [39] used eye-tracking technology to assess attention by measuring fixation duration, fixation counts, and eye movements to investigate the influence of front-of-pack (FOP) variations on consumer attention. The findings showed that FOP can attract the attention of respondents. Visual saliency is usually defined as the physical attributes of an object, such as color, shape, and movement, that catch respondents’ attention. This study provided a simple and cost-effective packaging design solution that uses eye tracking to eliminate cluttered information, attract the attention of consumers, and thus take a critical step toward the purchase decision [39]. Wu’s [53] research used the recording of fixed count and fixed time to study how the primary navigation (novel versus ordinary) of the apparel e-commerce influenced consumer attention, novelty perception, arousal, and approach behavior. The results showed that novel primary navigation leads to increased attention, novelty perception, arousal, and an increased willingness to approach the online store. Novelty perception and arousal serially mediated the effect of primary navigation on approach behavior. Atmospheric responsiveness plays a moderating role in the relationship between primary navigation and consumer approach behavior. The study provided online store owners with management insights into the design of major navigation and targeted optimization for webpage design [53].

García-Madariag et al. (2019) employed multimodal biosensors and multiple software tools combining eye tracking and electroencephalography (EEG) to measure attention. Declarative testing was utilized to assess preferences, and the findings suggested that implicit monitoring based on EEG and gaze data could predict the perception of package design colors [34].

Furthermore, ten studies supplemented their eye-tracking experiments with questionnaires and in-depth interviews, integrating eye-tracking data with subjective assessments. The dimensions measured included consumers’ preferences for multiple product types when choosing a product and the perceived importance of product attributes, such as price, flavor, brand, healthfulness, convenience, and ingredients. Examination of gender differences in visual attention and attitudes toward different types of advertisements revealed differences between men’s and women’s visual attention to images and their attitudes toward the loudness of the advertisements. Evaluating full-body versus partial-body graphic labels on plastic beverage bottles yielded the conclusion that consumers chose partial-body labels more frequently than full-body labels, regardless of the flavor of the beverage.

Overall, the reviewed investigations delved into consumers’ gaze behaviors, choice patterns, and design preferences, often with a focus on specific product variations. The insights garnered from such studies serve as valuable inputs for crafting designs that effectively capture the visual attention of consumers.

### 4.2. Application of Eye-Tracking System for Design Evaluation

In the application of eye-tracking technology in visual communication and new media design evaluation, two primary categories of eye-tracking systems were observed: screen-based and wearable eye trackers.

Table 7 presents a description of the two types of systems, including their strengths, limitations, and applications in the design evaluation in the selected studies.

Screen-based or wearable eye trackers are mostly used in label and package design studies, where they are instrumental in evaluating the intricate relationship between various labeling elements (e.g., organic and origin labels, pricing information, nutritional content) and package attributes (e.g., packaging materials, illustrations, and color schemes). In the field of advertising design, the primary focus is on evaluating the attention-grabbing elements of advertisements that resonate with consumers. Researchers in this domain explore disruptive elements within sustainable marketing practices and examine various aspects of advertising elements, including ad formats, celebrity endorsements, advertising logos, and slogans [30,43,47]. These studies offer insights into the elements that effectively attract consumer attention and contribute to the effectiveness of advertising campaigns.

Furthermore, eye-tracking studies examine the characteristics of websites and interfaces, exploring factors such as list versus matrix layouts, novelty versus familiarity, simplicity versus complexity, and consumers’ novelty perception, arousal, and proximity behaviors. These investigations shed light on the design features that significantly affect consumer attention, guiding the creation of more effective and engaging digital interfaces. Compared to traditional media, interface design is an interactive medium that relies heavily on the role of computers and focuses on customer experience.

Overall, eye-tracking technology plays an important role as an evaluation tool in the field of design research. It enables researchers to deeply analyze the association between design elements and consumers’ reflections, which in turn provides a more informed and effective basis for design strategy development.

### 4.3. Applications in the Design Domain

This literature review categorized the applications of eye-tracking technology into two design domains: visual communication design and new media design. By incorporating eye-tracking technology into design evaluation studies, one can proactively identify the elements that attract consumers’ attention and distinguish them from those that do not. These insights can enhance all aspects of design, including packaging design, advertising design, interface layout planning, and point-of-sale displays. To this end, this study delved into the utilization of eye-tracking technology in design and explored its innovative potential across diverse domains, including visual communication design and new media design.

Visual communication design mainly uses visual symbols to convey information, such as text, logos, and illustrations. Its subdivisions include packaging design, display design, advertising design, illustration design, and logo and font design. This integrated study reviewed the subdivisions of visual communication design, as shown in Table 8. In the field of visual communication design, the 30 papers in this study included 24 items in packaging design, advertising design, display, logo, and illustration design.

Packaging design is a systematic endeavor, with labels playing an integral role in the process. Eye-tracking research in the realm of advertising encompasses a multifaceted exploration of celebrity endorsements, slogans, and advertising texts, among others. In the realm of display design research, Drexler and Souček (2016) evaluated the cognitive processing of Generation Y consumers by examining the effect of dessert shelf positioning through eye-tracking experiments [33]. Conversely, Gomes et al. (2014) probed the influence of mannequin heads in physical store windows on consumer shopping behavior [35].

In the field of visual communication design, eye-tracking technology plays a vital role in assessing the effects of various design elements on consumer behavior. Specifically, it is often used to verify the influence of various design elements on factors such as attention allocation, product perception, purchase intention, and choice.

The new media design studies employ eye-tracking technology to investigate the effect of design components of multimedia web services and interface design on consumers’ visual behaviors. The incorporation of eye-tracking technology facilitates an examination of how different webpage elements, including navigation, visual complexity, and product listing page design, influence customer attention and reactions to products and advertisements while browsing websites. In evaluating the interface design components, analyzing the customer’s eye movements using eye-tracking technology can help identify design flaws that increase the objectivity of these evaluations. Table 8 illustrates the application of eye-tracking technology in new media design.

## 5. Discussion

### 5.1. Implications

Eye tracking is a well-established research tool for better understanding the eye movements and gaze of individual participants and sample populations [57]. A well-designed experiment yields coherent data, enables statistical analysis, and makes it possible to draw conclusions that are relevant to the examined context [56]. Eye-tracking technology can obtain a more objective evaluation of design elements than traditional questionnaires and interviews. In addition, eye tracking can capture the user’s gaze point and eye movement trajectory in real-time, which is particularly important for real-time user testing. This proves the versatility, real-time nature, and effectiveness of eye-tracking instruments in a wide range of design fields.

However, different eye trackers are tailored to specific research objectives. Screen-based eye trackers, frequently used in controlled laboratory settings, offer high precision and are ideal for testing multiple experimental stimuli in stable environments. In contrast, wearable eye trackers, such as Tobii glasses, more closely approximate natural behavior, allowing for the assessment of dynamic stimuli and participant movement, which is essential for real-world applications in physical environments. The naturalistic assessment provided by wearable systems adds ecological validity, making these findings more applicable to real-life design scenarios. Researchers need to carefully consider these distinctions when selecting the most appropriate eye-tracking system for their study objectives.

In design evaluations, screen-based eye trackers are often favored for visual communication and new media design because they can be easily utilized in controlled laboratory conditions. These conditions enable researchers to focus on specific eye movement measures, such as gaze direction and gaze point, which have been the primary variables in many studies. This level of precision is particularly useful in assessing user interactions with digital and physical interfaces, helping to optimize design elements for an enhanced customer experience.

Advanced eye-tracking technology offers additional metrics, including pupil diameter and dilation, eye position and identification, and saccades, which are often overlooked. For example, studies by Mikhailenko et al. (2022) and Kim and Lee (2021) highlighted the potential of pupil diameter as an indicator of emotional recognition, with observable correlations between emotional states and changes in pupil size. Despite this potential, few studies have actively incorporated pupil dilation data into their analysis, suggesting that focusing on this underexplored area could deepen our understanding of emotional responses and cognitive processing in design research [14,18].

This emphasis on gaze and fixation measures, while valuable, can provide only limited insights into eye-tracking technology in design evaluation. Broadening the metrics used in eye-tracking research by including saccades, pupil dilation, and blink rates could provide a more comprehensive understanding of both cognitive load and emotional engagement with design elements. Expanding the use of pupil-related metrics, saccades, and blinks alongside traditional measures could enhance our understanding of how consumers emotionally engage with design elements, providing a more holistic view of their interactions with visual stimuli. Therefore, future studies could benefit from integrating more diverse eye-tracking metrics to capture nuanced emotional and cognitive responses, contributing richer multidimensional insights into design research.

Furthermore, this study contributes to the theoretical understanding of design evaluation by positioning eye tracking as a critical tool for bridging the gap between cognition and the visual design effect. These insights can help refine existing models of consumer behavior and decision-making in response to visual stimuli, leading to the development of more accurate predictive models in both academic and applied research settings.

For practitioners, including designers, marketers, and user experience (UX) professionals, this study provides actionable insights into the application of eye-tracking technology in design evaluation. Eye-tracking data can inform the design process directly by identifying which elements capture user attention and which are overlooked, enabling the optimization of product packaging, advertising layouts, and user interfaces for better engagement. This technology allows practitioners to make evidence-based decisions that enhance user experience, drive consumer behavior, and increase brand impact.

### 5.2. Limitations and Suggestions for Future Research

This review identified several limitations and areas of improvement in the selected studies. First, the predominant reliance on university students and staff as samples limits the generalizability of the findings. Future research should expand participant diversity by including various age groups and occupational backgrounds to ensure broader applicability. Second, as technology and methodologies evolve rapidly, studies based on data from a specific period may not fully reflect current advancements. Longitudinal research that tracks trends and incorporates technological developments is essential for maintaining relevance. Moreover, excluding conference papers that often present innovative findings may result in biased conclusions. Future reviews should integrate conference papers to enhance their comprehensiveness and relevance.

A significant limitation is the inconsistency in the metrics used across studies, which affects the comparability of the findings. For example, key indicators such as pupil dilation, which is crucial for understanding cognitive and emotional effects, were often omitted. Future research should adopt standardized and diverse indicators to enable a holistic assessment of design impacts. Furthermore, the reviewed studies primarily relied on screen-based eye-tracking devices, with limited adoption of wearable devices and VR-based technologies. Wearable and VR eye-tracking tools offer significant advantages, such as capturing gaze behavior in naturalistic or immersive settings and enabling a more accurate analysis of attention, navigation patterns, and emotional responses [58]. This capability is particularly relevant for evaluating user experience in dynamic or virtual environments. Future studies should prioritize these technologies to enhance their ecological validity and deepen insights into user interactions.

The integration of multimodal biometric methods holds promise for advancing design research. Combining eye tracking with tools such as EEG, fNIRS, HRV, EMG, and ECG can provide richer insights into neurocognitive and emotional processes. This multidisciplinary approach enables a deeper understanding of how design elements influence behavior, perceptions, and emotions, thus supporting the development of sustainable user-centered designs. By analyzing emotional and physiological responses, researchers can bridge the gap between aesthetics, usability, and user satisfaction [59].

Lastly, the scope of this review focused on visual communication and new media design, yet eye-tracking applications extend to other domains, such as spatial and product design. Future studies should explore these areas to broaden the impact of eye-tracking research and uncover its potential in multidisciplinary contexts. Emerging technologies, including remote and online eye tracking, also warrant attention for their ability to expand research accessibility and flexibility, particularly in diverse real-world settings. In addition, wearable devices designed to track the eye movements of mobile observers have enabled the study of gaze behavior in more realistic naturalistic settings [60].

This review underscores the importance of integrating subjective methods, such as interviews and questionnaires, with objective eye-tracking data. This combined approach offers a robust framework for studying decision-making processes, many of which occur subconsciously while accounting for demographic and cultural diversity. Future research that builds on these recommendations can provide more comprehensive insights into the relationship between design and human behavior, advancing both theoretical understanding and practical applications.

## 6. Conclusions

In the field of behavioral sciences, understanding how visual design and media interaction influence consumer engagement is essential for companies aiming to expand their market reach. Research has increasingly focused on how consumers respond to visual and media elements with the aim of enhancing recognition and support through strategic design [61]. Eye-tracking technology has become a powerful tool in this field, offering precise insights into eye movements and gaze patterns across both individual and group samples. Well-constructed eye-tracking studies yield robust data, enable in-depth statistical analysis, and provide contextually rich insights that deepen our understanding of consumer behavior in real-world settings [56].

This review systematically explored the evolving role of eye-tracking technology in design assessment, with a particular focus on its growing relevance in visual communication and new media research over the past decade. While current applications have predominantly focused on identifying design elements that capture visual attention, this narrow emphasis on surface-level metrics limits our ability to understand the broader implications of design. To fully leverage the potential of eye-tracking technology, its application should be expanded beyond optimizing visual engagement to include multiple stages of the design process, such as conceptualization, prototyping, and comprehensive evaluation. This broader application can reveal how design decisions shape customer experiences in more intricate and meaningful ways, particularly in fostering sustainable and customer-centered behaviors.

Expanding design evaluation methods to incorporate a broader range of biometric measures will enhance the precision of design research and contribute to a deeper understanding of consumer-oriented behavioral experiences. By broadening the tools and methodologies used in design evaluation, we can better meet diverse customer needs and support more informed decision-making processes. By adopting such advanced methodologies, future research could transform the evaluation of visual and media designs, highlighting the critical role of evidence-based practices in creating engaging, sustainable, and customer-centric environments.

## Figures and Tables

**Table 1:** Classification of Search Terms.

OR		Methodology	Related Communication Design Areas	Related New Media Design Areas	Specification of Variables
		Eye tracking experiment	Visual communication	New media design	Design evaluation
			Graphic design	Web design	Customer experience
			Advertising design	UI design	Consumer behavior
			Display design		Visual perception
			Packaging design		Visual attention
			Product design		

**Table 2:** Inclusion and Exclusion Criteria.

Criterion	Inclusion	Exclusion
Topic	experimental	Not related to design
Method	Design-related	Non-experimental
Intervention		Not related to design No comparators
Data		Not responding to the PICO criteria Case of eye tracking

**Table 3:** Design discipline and type classification.

Author (Year)	Title	Design Disciplines	Design Types
Ballco et al. (2019) [27]	Consumer preferences for nutritional claims: An exploration of attention and choice based on an eye-tracking choice experiment	Visual communication design	Label
Barbierato et al. (2023) [28]	Wine label design proposals: An eye-tracking study to analyze consumers’ visual attention and preferences	Visual communication design	Label
Barbosa et al. (2021) [29]	Positioning of design elements on the packaging of frozen convenience food and consumers’ levels of attention: An experiment using pizza boxes	Visual communication design	Package
Boscolo et al. (2021) [30]	Gender differences: Visual attention and attitude toward advertisements	Visual communication design	Advertisements
Centurión et al. (2019) [31]	Relative Impact of Nutritional Warnings and Other Label Features on Cereal Bar Healthfulness Evaluations	Visual communication design	Label
De Keyzer et al. (2021) [32]	The processing of native advertising compared to banner advertising: An eye-tracking experiment	Visual communication design	Advertisements
Drexler et al. (2016) [33]	The influence of sweet positioning on shelves on consumer perception	Visual communication design	Display
García-Madariaga et al. (2019) [34]	Do isolated packaging variables influence consumer’s attention and preferences?	Visual communication design	Package
Gomes et al. (2014) [35]	The Effect of Full-Body Versus Partial-Body Graphic Labeling on Beverage Packaging	Visual communication design	Label
Hazuchová et al. (2018) [36]	Attention analysis of honey jar labels using eye-tracking techniques	Visual communication design	Label
Im et al. (2021) [37]	Beyond visual clutter: The interplay among products, advertisements, and the overall webpage	New media design	Webpage
Katz et al. (2019) [38]	Local and Organic Preference: Logo versus text	Visual communication design	Logo and text
Lacoste-Badie et al. (2020) [39]	Small change, big change—Increasing attention to product package variations	Visual communication design	Package
Lindström et al. (2016) [40]	Does the presence of a mannequin head change shopping behavior?	Visual communication design	Display
Liu et al. (2018) [41]	Effects of banner Ad shape and the schema creating process on consumer internet browsing behavior	Visual communication design	Advertisements
Liu et al. (2019) [42]	Good Slang or Bad Slang? Embedding Internet Slang in Persuasive Advertising	Visual communication design	Advertisements
Lourenção et al. (2020) [43]	Destination advertisement semiotic signs: Analyzing tourists’ visual attention and perceived ad effectiveness	Visual communication design	Advertisements
Merdian et al. (2021) [44]	Looking behind eye-catching design: An eye-tracking study on wine bottle design preference	Visual communication design	Package
Meyerding et al. (2018) [45]	Consumer preferences for organic labels in Germany using the example of apple’s—Combining choice-based conjoint analysis and eye-tracking measurements	Visual communication design	Label
Modi et al. (2023) [1]	Understanding Online Consumer Behavior at E-commerce Portals Using Eye-Gaze Tracking	New media design	Homepage
Nemergut et al. (2020) [46]	Influence of packaging attributes on the perception of juice: An eye-tracking study	Visual communication design	Package
Pelau et al. (2022) [47]	Celebrity vs. product: A Neuroscientific Approach to the Distractors in Food Advertising for Sustainable Marketing	Visual communication design	Advertisements
Peschel et al. (2019) [48]	Increasing consumers’ attention capture and food choice through bottom-up effects	Visual communication design	Illustration
Schmutz et al. (2010) [49]	Designing product listing pages-Effects on sales and users’ cognitive workload	New media design	Webpage
Vu et al. (2016) [50]	Design factors influence consumers’ gazing behavior and decision time in an eye-tracking test: A study on food images	Visual communication design	Illustration
Wang et al. (2017) [51]	Does a big Duchenne smile really matter on e-commerce websites? An eye-tracking study in China	New media design	Webpage
Wook et al. (2013) [52]	Exploring the effect of the human brand on consumers’ decision quality in online shopping: An eye-tracking approach	Visual communication design	Illustration
Wu et al. (2022) [53]	Navigating in online stores: The effect of the primary navigation on consumers’ response-A study based on the apparel e-retailer	New media design	Navigation
Xiao et al. (2023) [54]	Mobile marketing interface layout attributes that affect user aesthetic preference: an eye-tracking study	New media design	Interface
Yu et al. (2022) [55]	Why display motion on the packaging? The effect of implied motion on consumer behavior	Visual communication design	Package

**Table 4:** Participants.

Author (Year)	Sample Size	M	F	Mean Age	Condition
Ballco et al. (2019) [27]	100	49	51	-	Residents of a medium-sized town representative of Spain
Barbierato et al. (2023) [28]	60	29	31	-	A master’s student in the marketing program at the University of Florence
Barbosa et al. (2021) [29]	90	49	41	24	Engineering, Business, Design and Hospitality Management students at the Federal University of Pernambuco
Boscolo et al. (2021) [30]	180	90	90	age range 18~42	Students at a public university in Brazil
Centurión et al. (2019) [31]	100	25	75	age range 18~56	Students and staff of the Uruguayan Psychological Institute
De Keyzer et al. (2021) [32]	90	38	52	18~40	Belgian students and staff
Drexler et al. (2016) [33]	55	18	37	age range 20~30	Generation Y
					The temperature in the lab room is 21 degrees
García-Madariaga et al. (2019) [34]	40	19	21	22.8	University students
Gomes et al. (2014) [35]	28	7 (1st group)	7 (1st group)	age range 21.7	-
		7 (2nd group)	7 (2nd group)	age range 37	
Hazuchová et al. (2018) [36]	35	17	18	age range 20~35	-
Im et al. (2021) [37]	90		90	age range 18~62	University students
Katz et al. (2019) [38]	88 (1st group)	-	-	median age 37	High household income earners
	81 (2nd group)	-	-	median age 44	
	86 (3rd group)	-	-	median age 38	
Lacoste-Badie et al. (2020) [39]	165	15	150	26.95	-
Lindström et al. (2016) [40]	79	0	79	24.7	-
Liu et al. (2018) [41]	138	73	65	21.25	Experienced in the use of the internet
Liu et al. (2019) [42]	120	49	71	22.42	Participants in the MBA program
					Those with independent income and years of work experience
Lourenção et al. (2020) [43]	97			age range 18~40	Undergraduate and graduate students
Merdian et al. (2021) [44]	37	27	10	46.46	Highly educated people
Meyerding et al. (2018) [45]	73	38	35	34.9	Those who graduated from the University of Applied Sciences
					High-income group
Modi et al. (2023) [1]	76	33	43	-	-
Nemergut et al. (2020) [46]	38	16	22	24.53	Generation Y
Pelau et al. (2022) [47]	24 (1st session)	-	-	age range 19~25	-
	19 (2nd and 3rd session)			age range 19~25	
Peschel et al. (2019) [48]	127	55	72	age range 18~24	University students
Schmutz et al. (2010) [49]	20	-	-	22.4	-
				23.5	
Vu et al. (2016) [50]	100	50	50	age range 18~53	Students and staff of the Vienna University of Natural Resources and Life Sciences (BOKU)
Wang et al. (2017) [51]	52	0	52	21.7	A major university in southern China
Wook et al. (2013) [52]	38	16	22	-	University students
Wu et al. (2022) [53]	46	-	-	-	University students
Xiao et al. (2023) [54]	37	18	19	24.35	All participants have experience in marketing activities
Yu et al. (2022) [55]	63	22	41	age range 18~56	Current students and alumni in different educational fields

**Table 5:** Intervention and Comparator.

Author (Year)	Intervention	Comparator	Stimulus
Ballco et al. (2019) [27]	Yogurt types and variables: - Additive-free yogurt, fruit-free, grain-fiber yogurt	Compare different types of yogurts	Product images
Barbierato et al. (2023) [28]	Wine label illustration design options and variables: - Costumes (Sardinian folk costumes), oleaster Sardinian symbols (Sa Pintadera), and symbols (Wind-blown oleaster)	Wine label illustration design options	Wine bottle images
Barbosa et al. (2021) [29]	Packaging page layouts and variables: - Images, logo, flavor, and quick product information (top right, bottom left, bottom right and bottom left)	Packaging page layouts of comparison	Product images
Boscolo et al. (2021) [30]	Types of gender-specific product ads and variables: - Male products, female products, and neutral product advertising (product images, branding, text and slogans)	Visual elements of gender-specific product advertisements of comparison	Product advertisement
Centurión et al. (2019) [31]	Nutritional claims labeling characteristics and variables: - Images of fruit, nutrition claims for fiber content, nutrition warnings for excessive sugar and saturated fat content (with and without)	Nutritional claim labeling characteristics of the comparison	Cereal bar label image
De Keyzer et al. (2021) [32]	Ad types and variables: Native ads and banner ads (familiar, unfamiliar)	Ad types of comparison	Facebook page
Drexler et al. (2016) [33]	Shelf layout and variables: packaging, brands and prices	Comparative shelf layout	Shelf layout image
García-Madariaga et al. (2019) [34]	Product packaging and variables: - Images (no images, logos, and product context images). - Text (no text, positive and negative) - Colors (neutral, cool, and warm)	Packaging visual elements of comparison	Product images
Gomes et al. (2014) [35]	Drink types and variables: - Water, mixed berries, green tea, orange juice, coffee and lemon sports energy drinks	Drink types of comparison	Authentic shopping environment: 12 beverage bottles
Hazuchová et al. (2018) [36]	Packaging materials and variables:Glass packaging and plastic packaging	Packaging materials of comparison	Product pictures
Im et al. (2021) [37]	Product pages and variables: - Product page complexity (layout, color scheme, presentation order)	Product pages of comparison	Web page
Katz et al. (2019) [38]	Product types and variables: - Local product and organic product (logo and text label)	Product types of comparison	Product images
Lacoste-Badie et al. (2020) [39]	Layout of visual elements and variables on the front package: Same front-of-pack (FOP) layout (horizontal arrangement, vertical arrangement), FOP layout with differences (brand mix, text message mix, color mix)	Layout of visual elements of comparison	Shelf map with target products
Lindström et al. (2016) [40]	Window mannequins and variables: With and without heads	Window mannequins of comparison	Photos of mannequins
Liu et al. (2018) [41]	Ad shapes and variables: Diamond banner ads and rectangular banner ads	Ad shapes of comparison	Sites with banner ads
Liu et al. (2019) [42]	Product advertising copywriter and variables: Advertising copy for necessities and advertising copy for luxury goods (in three different languages: SL, ESL, and EIL)	Comparing product advertising copy in different languages	Advertising copywriter
Lourenção et al. (2020) [43]	Destination advertising attributes and variables: Induced communication and combined communication (brand logos, slogans) in advertising	Destination advertising attributes of comparison	Advertising images
Merdian et al. (2021) [44]	Wine product categories and variables: - Minimalist, thematic, standard, and premium (red, white, interesting, valuable)	Wine product categories of comparison	Product images
Meyerding et al. (2018) [45]	Product labeling visual elements and variables: Organic labeling, origin, and price	Product labeling visual elements of comparison	Product images
Modi et al. (2023) [1]	Site pages and variables: Home page, product search page, product details page	Site pages of comparison	Website home screen
Nemergut et al. (2020) [46]	Packaging visual characteristics and variables (normal saturation, desaturation, front message, back message; packages with images of real oranges, and packages with illustrations of oranges)	Packaging visual characteristics of comparison	Juice packaging pictures
Pelau et al. (2022) [47]	Types of advertisements and variables: Two different advertisements for the same product (emphasizing the celebrity, emphasizing the product)	Types of advertisements of comparison	Collage
Peschel et al. (2019) [48]	Top-down organic labeling characteristics and variables:Tomato, chocolate, yogurt (small size/large size; low significance/high significance)	Top-down organic labeling characteristics of comparison	Organic labeling
Schmutz et al. (2010) [49]	Web page categories and variables: Matrix and list modes	Web page categories of comparison	Product page mode
Vu et al. (2016) [50]	Illustration design factors and variables: - Number of images (two, three, four, five, and six). - Questioning content (five dimensions of food: taste, health, price, convenience, and familiarity). - Types of assessment (five types of assessment: maximum choice, minimum choice, ranking, rating, and grouping)	Illustration design factors of comparison	Food images
Wang et al. (2017) [51]	Web advertising mannequin expression characteristics and variables: Duchenne smile (strong, weak), non-Duchenne smile (strong, weak)	Web advertising mannequin expression characteristics of comparison	Perfume page with model’s expression
Wook et al. (2013) [52]	Characteristics of celebrity-endorsed advertising and variables: Functional and symbolic products (celebrities, texts, logos)	Comparison of celebrity endorsement advertising features	Online shopping brand product screen
Wu et al. (2022) [53]	Web site main navigation features and variables: Novel and common (twelve seasons)	Web site main navigation features of comparison	Web image
Xiao et al. (2023) [54]	Page layout properties and variables: Low and high symmetry, high and low simplicity	Page layout properties of comparison	Interface images
Yu et al. (2022) [55]	Front package illustration features and variables: One with an image of the product in motion (implied motion condition) and the other with a static image of the product (no implied motion condition)	Front package illustration features of comparison	Product images

**Table 6:** Outcome.

Author (Year)	Eye Tracker Tool	Type	Indicator Measurements	Outcome	Analysis Method
Ballco et al. (2019) [27]	Screen-based eye trackers	Tobii X2-30ET	Fixed count and fixed time	Attention and Preferences	*t*-test, ANOVA F-test, and Bonferroni post-hoc test, Econometric analysis, questionnaire analysis
Barbierato et al. (2023) [28]	Pupil labs wearable eye- tracking	Pupil invisible glasses	Fixed count and time to first focus	Attention and Preferences	Data visualization (heat map) and regression analysis
Barbosa et al. (2021) [29]	Screen-based eye trackers	Tobii X-120	Fixed count	Attention	*t*-test; Poisson and negative binomial models, descriptive statistics
Boscolo et al. (2021) [30]	Screen-based eye trackers	Tobii studio (NR)	The average total duration of gaze	Attention, Advertising Attitude	*t*-test; Heat map analysis; questionnaire analysis;
Centurión et al. (2019) [31]	Screen-based eye trackers	Tobii T-60	Time to the first fixation, fixation count	Health Assessment, Health Perception	ANOVA, Tukey test, generalized linear model
De Keyzer et al. (2021) [32]	Screen-based eye trackers	Tobii pro tx3000	Total focus duration, focus count, average visit duration	Attention	*t*-test, Hayes’ PROCESS macro
Drexler et al. (2016) [33]	Screen-based eye trackers	SMI RED 500	Dwell time, hit ratio, and revisitors	Purchase decision	Heat map analysis, statistical analysis, in-depth interview
García-Madariaga et al. (2019) [34]	Screen-based eye trackers	Tobii X20-30	Focus count, viewing time, the time it takes the user to reach each area	Attention, Preferences	ANOVA, Kruskal-Wallis H-test, questionnaire analysis
Gomes et al. (2014) [35]	Wearable eye tracking	Tobii T60	Fixation count, time to first fixation, visit count, fixation duration	Preferences	ANOVA
Hazuchová et al. (2018) [36]	Screen-based eye trackers	SMI RED 250	Order, entry time, stay time, hit rate, return, Returner average gaze, first gaze, number of gazes	Behavior, Perception, Attention	KPI analysis, in-depth interview
Im et al. (2021) [37]	Screen-based eye tracker	Tobii T-60	Total focus, total focus duration	Chemical reaction	ANOVA, questionnaire analysis
Katz et al. (2019) [38]	Screen-based eye trackers	Tibii X1 light	Time to first focus (TFF), first focus duration (FFD), total visit duration (TVD), focus count (FC)	Preferences	*t*-test
Lacoste-Badie et al. (2020) [39]	Screen-based eye trackers	SMI RED 250	Fixed time, fixed count	Attention	ANOVA
Lindström et al. (2016) [40]	Screen-based eye trackers	High-Performance Camera	Number of observations, length of observation	Browsing behavior	*t*-test
Liu et al. (2018) [41]	Screen-based eye trackers	ASL-D6	Fixed time, fixed count, pupil diameter	Advertising attention, brand awareness, product reviews, and advertising attitudes	ANOVA, post-hoc tests
Liu et al. (2019) [42]	Wearable eye tracking	Tobii (NR)	Total visit duration	Purchase intent	ANOVA, mediation analysis
Lourenção et al. (2020) [43]	Screen-based eye trackers	Tobii X1L	Number of views in the total advertised area, number of views in the area of interest, duration of views in the total advertised area, duration of views in the area of interest	Visual attention, perceived advertising effectiveness	ANOVA, questionnaire analysis
Merdian et al. (2021) [44]	Screen-based eye trackers	Tobii pro x120	Time to first duration, first focus duration	Attention	ANOVA
Meyerding et al. (2018) [45]	Wearable eye tracking	Tobii Pro Glasses 2	Fixation duration, fixation count, visit duration, visit count	Attention and preferences	Conjoint analysis, heatmap analysis
Modi et al. (2023) [1]	Screen-based eye trackers	-	Fixed count and time to first focus	Consumer behavior	*t*-test;
Nemergut et al. (2020) [46]	Screen-based eye trackers	SMI RED 250	The average length of stay	Concerns and favorites	*t*-test, Mann-Whitney, in-depth interview
Pelau et al. (2022) [47]	Screen-based eye trackers	Not further specified	Total gaze entry time	Attention	descriptive statistics and discriminant analysis
Peschel et al. (2019) [48]	Screen-based eye trackers	Tobii T60 XL	Fixation likelihood	Attention and choice	SAS 9.3, binomial response distribution. a logit link function
Schmutz et al. (2010) [49]	Screen-based eye trackers	Tobii 1750	First focus duration, fixed quantity, fixed length	Cognitive load decision-making	*t*-test, ANOVA
Vu et al. (2016) [50]	Screen-based eye trackers	Tobii T60	Fixed time, fixed count, visits fixed time, visit fixed time	Attention to behavior Decision time	ANOVA, questionnaire analysis
Wang et al. (2017) [51]	Screen-based eye trackers	SMI RED 500	Fixed count, fixed time	Attention purchase intention	*t*-test, ANOVA, questionnaire analysis
Wook et al. (2013) [52]	Screen-based eye trackers	Tobii x120	Focus duration	Sense-making	ANOVA, heat map analysis, questionnaire analysis
Wu et al. (2022) [53]	Screen-based eye trackers	Tobii T60 XL	Fixed count, fixed time	Novelty perception, arousal, and proximity behavior	*t*-test, questionnaire analysis
Xiao et al. (2023) [54]	screen-based eye tracker	Tobii Pro X3-120	Fixed count, fixed time, pupil diameter	Aesthetic preference	*t*-test, ANOVA
Yu et al. (2022) [55]	Screen-based eye trackers	SMI RED 250	Fixed count, fixed time, dwell time, first focus duration	Attention, Product evaluation, Purchase intention, and choice	*t*-test, Logistic regression analyses, regression analysis, questionnaire analysis

**Table 7:** Overview of Eye-tracking System for Design Evaluation.

	Description	Strengths	Limitation	Design Evaluation	References
Screen-based	Feature non-intrusive eye tracking. Require a computer or screen installation. The respondents need to sit in front of the ET and calibrate before the experiment. Record distant eye movements.	Observations or offline stimuli of any screen-based stimulus material can be recorded.	Require relative fixation of the subject’s head.	Visual communication design (label design/packaging design/advertising design New media design (web design)	[27,29,30,31,32,33,34,35,36,37,38,41,42,43,44,46,47,48,49,50,51,52,53,54,55]
Wearable	They are mounted onto lightweight glasses. Record eye movements from close range.	Respondents could walk around. Their head position can be unfixed, and calibration is not necessarily required. Do research in real-world settings.	Can only record eye movements up close.	Visual communication design (label design/packaging/display)	[28,35,40,45]

**Table 8:** Application in Visual Communication Design and New Media Design.

Researcher (Year)	Purpose	Method (Type of Eye Tracker)	Key Findings
Ballco et al. (2019) [27]	To assess consumers’ preferences for multiple NCs (nutritional claims) when choosing yogurt, and to explore the relationship between NC preferences and visual attention to these claims.	Screen-based	Low-sugar NC was the least popular claim of all the models. The presence of NCs generally increased visual attention in terms of fixation times, which may be positively associated with the likelihood of influencing the final decision to purchase NC-containing yohurt.
Barbierato et al. (2023) [28]	To examine the relationship between consumers’ visual attention to wine label design and their preferences.	Wearable	There is a strong positive correlation between a particular design option and the explicit choice of the same bottle. Labels with clothing embellishments can encourage consumers to buy and are the most popular design options.
Barbosa et al. (2021) [29]	To explore how the positioning of design elements affects consumers’ attention to convenience food packaging.	Screen-based	The positioning of the image, logo, and flavor specification elements affected the attention levels. However, no effect for quick information was found.
Boscolo et al. (2021) [30]	To examine gender differences in visual attention and attitudes toward different types of advertising.	Screen-based	For women’s advertisements, the difference between visual attention and attitude was insignificance. However, significant differences emerged in men’s visual attention to images and their relative attitudes to advertisements. Men and women differed in visual attention, e.g., car images were the most popular with men.
Centurion et al. (2019) [31]	To investigate the relative influence of nutritional warnings and two marketing strategies commonly used in food labels, nutrient claims, and fruit images on consumers’ healthfulness judgments.	Screen-based	Nutritional warnings captured participants’ attention and reduced the visual attention required to assess health; overall, participants relied on them to make health judgments.
De Keyzer et al. (2021) [32]	To study the effect of advertising format (banner versus native) on visual attention.	Screen-based	Native ads attracted more and longer visual attention than banner ads. Longer visual attention increased persuasive knowledge and recognition of advertising, leading to improved brand recognition.
Drexler et al. (2016) [33]	To find out how people perceive the positioning of various shelf elements and confectionery.	Screen-based	The most important factors were the price and discount of the product, but customer habits and experience also mattered. Men focused their attention on the middle of the shelf; women behaved differently, paying attention to every element and every commodity on the shelf. The idea of candy as a product had a great influence on the attention of consumers.
Garcia-Madariage et al. (2019) [34]	To examine the effect of different packaging attributes on consumers’ attention levels.	Screen-based	The presence of visual elements (images or text on the packaging) improved the attention of the participants. Color modification had no significant effect on participants’ neurophysiological attention levels. Participants’ neurotheological effects did not necessarily coincide with their subjective assessments of preferences.
Gomes et al. (2014) [35]	To examine and assess the shelf presence of full-body graphic labels versus partial-body graphic labels on plastic beverage bottles by collecting quantitative and qualitative data.	Wearable	Both label sizes attracted an equal amount of visual attention. However, consumers chose partial-body labels more often than full-body labels, regardless of the beverage’s flavor or age group.
Hazuchova et al. (2018) [36]	To explore consumer behavior, focusing on the effects of packaging and labeling on young consumers aged 20–35, inquiring about whether and to what extent packaging and labeling affect honey purchases.	Screen-based	The most noticeable aspect of honey packaging was the brand of the producer, as well as the variety description and name of the honey. The least noticed was the weight detail and graphic design of the packaging.
Katz et al. (2019) [38]	To examine consumer preferences for local and organic products, particularly the effect of logo and text label formats.	Screen-based	Consumers preferred local products over non-local products. Native logos tended to grab attention faster and held it longer than text labels. Some consumers preferred organic text labels over the SUDA-certified organic logo.
Lacoste-Badie et al. (2020) [39]	To investigate the effect of front-of-package (FOP) changes on consumers.	Screen-based	In a cluttered retail environment, front-of-pack variations could draw consumer attention. Changes in FOP caught the attention of respondents.
Lindström et al. (2016) [40]	To determine whether the presence of a mannequin head influences consumers’ purchase intentions for displayed merchandise.	Wearable	In brick-and-mortar stores, the presence of humanized heads enhanced purchase intent for items displayed on mannequins.
Liu et al. (2018) [41]	To provide evidence for the process of creating new advertising formats by measuring the physiological behavior of participants.	Screen-based	Banner shapes that did not align with consumers’ existing patterns generated more browsing behavior than when the shape was consistent. After additional exposure to the new shape, browsing behavior decreased significantly compared to the first exposure.
Liu et al. (2019) [42]	To examine the effects of internet slang characteristics on advertising attention, brand awareness, product reviews, and advertising attitudes.	Screen-based	The use of internet slang in advertisements significantly increased consumer attention compared to standard language but did not necessarily increase product reviews and brand awareness for all types of goods.
Lourenção et al. (2020) [43]	To analyze the effect of the type of dissemination of destination photos and destination country brand logos and slogans used in advertisements on tourists’ visual attention and perceived advertising effectiveness is analyzed.	Screen-based	Destination photos provided by inductive communication had a more positive effect on tourists’ perceived advertising effect than combined communication. The destination country brand logo and slogan drew greater visual attention of tourists to the whole advertisement.
Merdian et al. (2021) [44]	To explore how unconscious perception differs from conscious response when evaluating a wine bottle on a shopping shelf.	Screen-based	Compelling design did not automatically translate into higher value perception and interest in a product. The standard bottle was attributed the highest value and highest interest in the experiment. Thematic and atypical wines were most likely to be attributed to the ‘striking’ description.
Meyerdin et al. (2018) [45]	To examine German consumer preferences for organic labeling in the case of apples.	Wearable	Consumers who derived greater utility from specific attributes of goods also paid greater visual attention to them. Combining stated preference methods with eye-tracking techniques had the potential to address some of the major limitations, such as social desirability, memory limitations, and lock of visual focus on specific product features. The highest partial value utility was the ‘local’ specification, followed by domestically produced apples with the specification ‘Germany’, with ‘local’ and ‘Germany’ getting the highest values (preferences).
Nemergut et al. (2020) [46]	To explore how different attributes of packaging can affect customer perceptions of juice.	Screen-based	Lower saturation significantly reduced the focus on individual packages compared to brighter colors and reduced the effect on juice cravings. Photography of oranges increased the cravings for the juice compared to illustrations of oranges.
Pelau et al. (2022) [47]	To explore the interference factors of celebrity endorsement in sustainable marketing in product advertising.	Screen-based	The celebrity was the eye-catcher of the ad and is the first and longest-viewed object, but products and logos are also viewed. The number of objects in an advertisement affected consumers’ attention. In the case of information overload, participants tended to watch the most familiar elements of celebrity endorsements.
Peschel et al. (2019) [48]	To explore the question of how individual labels on product packaging can attract attention through bottom-up effects and increase choice through increased attentional attraction.	Screen-based	Attention capture increased significantly for large, more visually salient labels.
Vu et al. (2016) [50]	To investigate how test design factors affect food consumers’ gaze behavior and decision timing.	Screen-based	Review and number of images, but not question content, significantly influenced gaze behavior and decision time.
Wook et al. (2013) [52]	To analyze visual attention through eye-tracking technology to determine how the use of human branding affects the quality of consumer decision-making in an online shopping environment.	Screen-based	The adoption of human brands in online stores influenced consumers’ perceived decision quality. The results supported a significant difference in perceived product trust between the two levels of perceived decision quality. Product type affected consumers’ perceived trust in products. Individuals placed more trust in functional products than in symbolic products.
Yu et al. (2022) [55]	To explore the effect of implied motion as a package design technique on consumer attention, product evaluation, purchase intention, and choice.	Screen-based	Implied motion significantly increased visual attention, resulting in more frequent choices. Implied motion can enhance freshness, which translates into better taste, product appeal, and ultimately increased purchase intent.
Im et al. (2021) [37]	To investigate how consumers divide their attention and evaluate products and advertisements on complex web pages while casually browsing the web.	Screen-based	Participants developed an overall impression of the complexity of the overall web page, and this impression spilled over into evaluations of individual elements on the web page (e.g., products, advertisements). An inverted U-shaped relationship (as opposed to a linear negative correlation) was observed between web page visual complexity and attitudes toward web pages, products, and advertisements.
Modi et al. (2023) [1]	To identify the most influential elements of a graphical interface and their effects on consumers’ visual attention.	Screen-based	Graphical interfaces attracted customer attention. Gender-specific visual preferences for various parts of the graphical user interface were observed.
Schmutz et al. (2010) [49]	To examine the effect of presentation formats, lists versus matrices on shopping decision quality and subjective mental load and studies attentional processes in both presentation modes.	Screen-based	List presentation was associated with lower cognitive load and more economical product selection.
Wang et al. (2017) [51]	To investigate the joint effect of a model’s smile type and intensity on consumers’ attention and purchase intention on an e-commerce website.	Screen-based	Products with a Duchenne smile increased purchase intention and attention from participants more than products without a Duchenne smile. Smile intensity moderated the effect of smile type on participants’ attention to packaging photos and product descriptions. When the intensity of the smile was low, Duchenne smiles attracted more attention to photos and descriptions than non- Duchenne smiles, whereas strong smiles had the opposite effect.
Wu et al. (2022) [53]	To examine how the primary navigation (novelty and commonplace) of an apparel e-retailer affects consumers’ attention, novelty perception, arousal, and approach behavior.	Screen-based	The primary navigation increased attention, novelty, arousal, and the willingness of users to approach the online store. Novelty perception and arousal continuously mediated the effect of primary navigation on approach behavior. In the relationship between online navigation and consumer approach behavior, atmospheric response played a leading role.

## Data Availability

Available upon request.
